# Implementation and evaluation of a group peer mentoring and leadership development program for research faculty in academic medicine

**DOI:** 10.1017/cts.2025.37

**Published:** 2025-03-26

**Authors:** Linda H. Pololi, Janet T. Civian, Mark Brimhall-Vargas, Vasilia Vasiliou, Arthur T. Evans, Kacy Ninteau, Lisa A. Cooper, Brian T. Gibbs, Robert T. Brennan

**Affiliations:** 1 National Initiative on Gender, Culture and Leadership in Medicine: C-Change. Institute for Economic and Racial Equity, The Heller School for Social Policy and Management, Brandeis University, Waltham, MA, USA; 2 School of Business, Clark University, Worcester, MA, USA; 3 Weill Cornell College of Medicine, New York, NY, USA; 4 Johns Hopkins School of Medicine, Baltimore, MD, USA; 5 UMass Memorial Health Care, Worcester, MA, USA

**Keywords:** Peer-mentoring, faculty, research, academic medicine, diversity, leadership

## Abstract

**Introduction:**

Research faculty often experience poor mentoring, low vitality, and burnout. We report on our logic model inputs, activities, measurable outcomes, and impact of a novel mentoring intervention for biomedical research faculty: the *C-Change Mentoring & Leadership Institute*. We present a) a detailed description of the curriculum and process, b) evaluation of the program’s mentoring effectiveness from the perspective of participants, and c) documentation of mentoring correlated with key positive outcomes.

**Methods:**

A yearlong facilitated group peer mentoring program that convened quarterly in person was conducted twice (2020–2022) as part of an NIH-funded randomized controlled study. The culture change intervention aimed to increase faculty vitality, career advancement, and cross-cultural competence through structured career planning and learning of skills essential for advancement and leadership in academic medicine. Participants were 40 midcareer MD and PhD research faculty, half women, and half underrepresented by race or ethnicity from 27 US medical schools.

**Results:**

Participants highly rated their mentoring received at the Institute. Extent of effective mentoring experienced correlated strongly with the measurable outcomes of enhanced vitality, self-efficacy in career advancement, research and work-life integration, feelings of inclusion in the program, valuing diversity, and skills for addressing inequity.

**Conclusions:**

The mentoring model fully included men and women and historically underrepresented persons in medicine and minimized problems of power, gender, race, and ethnicity discordance. The intervention successfully addressed the urgencies of sustaining faculty vitality, developing faculty careers, facilitating cross-cultural engagement and inclusion, and contributing to cultivating cultures of inclusive excellence in academic medicine.

## Introduction

With the intellectual and physical demands and emotional intensity of medicine, biomedical faculty need powerful commitment and high vitality; however, studies show low levels of vitality and inadequate mentoring [1–3] with high burnout and substantial attrition [4–6]. Research faculty report high rates of anxiety, depression, ethical and moral distress, and unprofessional behaviors [4,7,8]. About 40% of first NIH R01 grant recipients do not continue their careers with the support of federal funding [9]. Discrimination and sexual harassment on the basis of race, ethnicity, sexual orientation, and gender identity are commonplace in academic medicine institutions and intractable [10–17]. These findings suggest the need to change the culture of academic medicine, a complex undertaking that requires more effort and resources than most academic medicine centers are prepared to devote. Mentoring has been suggested as one strategy to address low vitality and high burnout, but only a third of faculty report having received good mentoring [2,3]. A recent scoping review of formal mentoring programs for health care faculty reported on different types of programs and recommended more rigorous program evaluation and investigation of effective components of program design [18].

Our own group peer mentoring program, the *C-Change Mentoring & Leadership Institute* (Institute), aims to provide effective mentoring, embody desired attributes of a positive and inclusive academic medical culture for all faculty, and meet the imperative of advancement in academic medicine of both well-represented and historically underrepresented racial and ethnic groups and women faculty. We recently reported the Institute’s positive impact in terms of vitality, self-efficacy, cross-cultural awareness, and valuing diversity, with similar findings among M.D. and Ph.D. men and women from both majority and underrepresented minority racial and ethnic groups from a randomized controlled study [19,20].

This study validates our program theory and activities by adding to the literature: a) an evaluation of the program’s mentoring effectiveness from the perspective of faculty participants, b) documentation of the participants’ experience of mentoring correlated with key positive outcomes in our randomized controlled study,[19] and c) a detailed description of the curriculum and process of the mentoring model (not previously published).

### Implementation and evaluation of a group peer mentoring and leadership development program for research faculty in academic medicine

*“[I found] a community of thoughtful fellows, sharing in the possibilities in our lives, as we ponder and chart our paths ahead. The intentionality of the process, to ask difficult questions, to put pen down to paper and flesh it out, with help from fellows and facilitators who listen, support and challenge. The luxury of taking a few days to step back from the daily tasks has shown how it is a necessity to pause, reflect and define our goals. The C-Change garden is filled with blossoms of many hues – the richness that comes from a group of people from different institutions, different areas of study, different geographic locations and different cultural backgrounds. [It] has contributed greatly to my own self-reflection, forcing me to ask questions that I have avoided and even figuring out what questions I need to ask. The Institute has nudged me along gently on a deliberative path that focuses on both the personal and the professional, as I seek meaning and fulfillment in both those areas of my life.”*

*– Faculty participant writing*

## Materials and methods

### Mentoring logic model, theoretical foundations, and approach

The Institute aspires to address faculty feelings of demoralization and isolation[1,21] by providing faculty with an opportunity to work and learn in a relational environment characterized by reciprocity rather than competition – one that is aligned with the necessity of collaboration and teamwork in science and medicine as is demanded by, for example, NIH Clinical Translational Science Awards (CTSA)[22] and superior medical practice.

### Inputs

**Figure 1.**
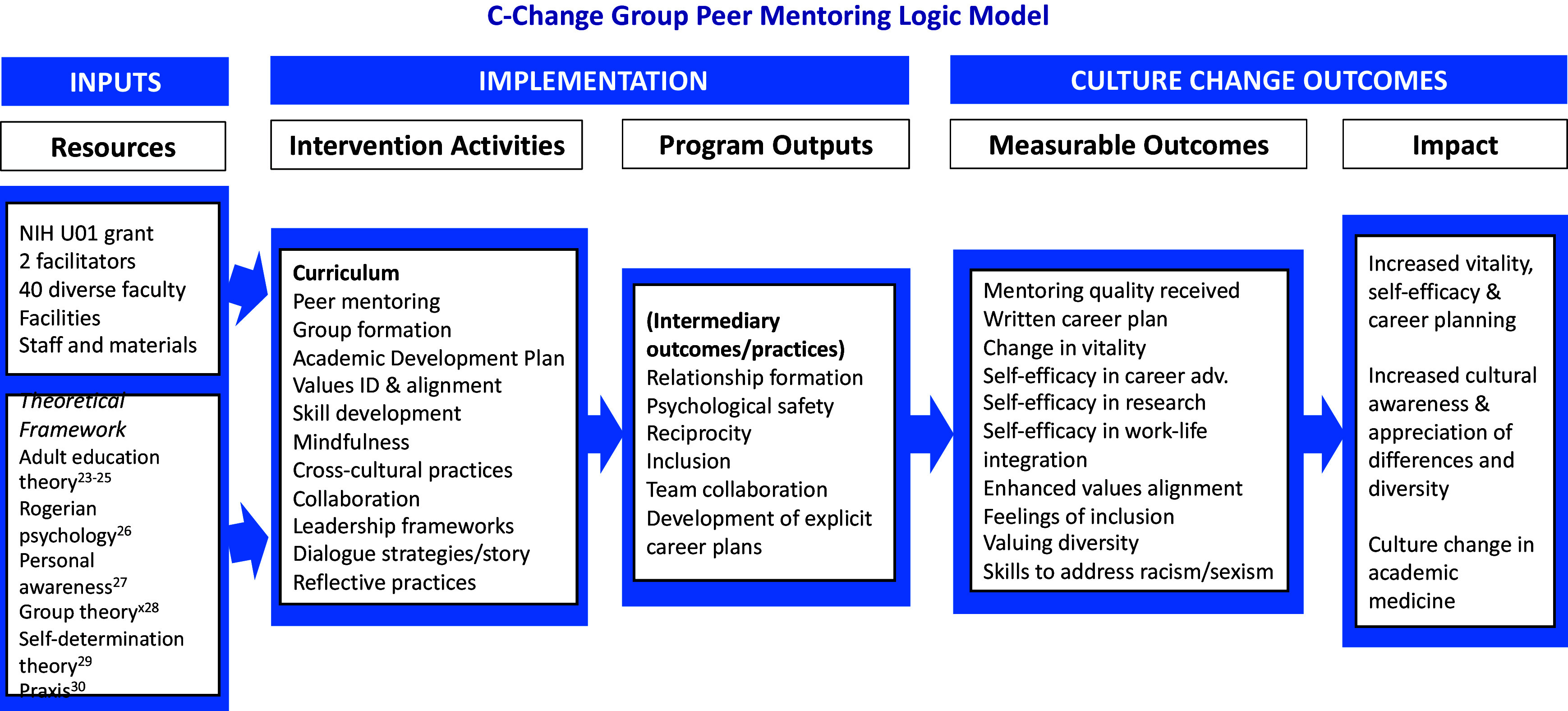
C-Change group peer mentoring logic model.

Figure 1 illustrates our logic model. The “inputs” included an NIH award to examine the efficacy and mechanisms of action at work in the mentoring model. The intervention was conducted twice (2020–2021 and 2021–2022). The mentoring model’s design used theoretical foundations of adult learning theory [23–25], Rogerian relational psychological principles [26], the need for developing personal awareness [27], group theory [28], self-determination theory [29], and praxis – when reflecting on learning enhances learning [30], and the benefits of working in demographically diverse groups [31–35].

Two experienced facilitators managed the timing and structure of the agenda throughout each day, maintaining a safe environment for everyone and ensuring space and time for all to be heard. Participants were provided with binders and printed handouts to accompany each aspect of the curriculum.

Each yearlong Institute convened four times on a quarterly seasonal basis for 2 or 3 days (total of 9 days, 80 hours). Participating faculty resided and convened in a secluded location free from distractions and work and home responsibilities. The sessions were designed for face-to-face implementation. Due to Covid-19 travel restrictions, the first two sessions of the initial cohort met by virtual conferencing. All other sessions were convened in person.

### Participating faculty recruitment

Participants in the program consisted of 40 early midcareer faculty in academic medicine who had demonstrated a substantial research focus in their careers (Table 1). To obtain the sampling frame for our RCT, NIH RePORTER was searched for all awardees receiving qualifying grants from 2013 to 2019. As our research design called for 50% participation by persons from underrepresented racial and ethnic groups as defined by NIH[31] (Black/African-American, Hispanic/Latinx, Native American, Alaska Native, and Pacific Islander), additional methods (previously described [19]) were used for focused recruitment. Invitations to apply were sent to over 5000 faculty. Of 270 applications received, 99 met all inclusion criteria: a) appointment for 3–14 years at a U.S. medical school or teaching hospital; b) associate professor or at least two years at rank of assistant professor (or equivalent); and c) demonstrated research that included a current or recent first-time NIH R01 or R01 equivalent award; R21 or R34 award; HRSA, ARHQ, or other federal agency major grant; K training grant; or recent major foundation or professional organization grant. We excluded those with more than one R01-equivalent award. In 2020, the recruited 99 early midcareer research faculty were randomly allocated to either the initial or delayed intervention group (20 participants per cohort), stratified by gender, degree, and race and/or ethnicity, or to similarly stratified waiting lists that would be used to fill any changes in enrollment prior to the start of the program. Participants came from 27 medical schools in 16 states.

**Table 1.** Demographic characteristics of two cohorts of 20 academic medicine midcareer research faculty participating in the C-Change group peer mentoring intervention in 2020–2021 and 2021–2022

Characteristics	Institute participants (n = 40)
	Number (%)
Female	20 (50)
Male	20 (50)
Race and ethnicity by gender	
Non-URM male	10 (25)
Non-URM female	10 (25)
URM male	10 (25)
URM female	10 (25)
Degree	
Ph.D.	18 (45)
M.D.	17 (43)
Both M.D. & Ph.D.	5 (13)
Rank	
Assistant professor	17 (43)
Associate professor	23 (58)
NIH research award	
K award recipients	19 (48)
R01 award recipients	15 (38)
Recipients of both K and R01 awards	5 (13)
Mean years in academic medicine in 2019 (SD)	8.9 (2.6)
Mean number of publications (SD)	29.2 (18.2)

URM: underrepresented in medicine. Individuals from racial and ethnic groups that have low representation in the health-related sciences and STEM fields on a national basis, as designated by the National Institutes of Health and the National Science Foundation. Non-URM: non-underrepresented in medicine. National Institutes of Health. November 22, 2019. Accessed July, 2024 https://grants.nih.gov/grants/guide/notice-files/NOT-OD-20-031.html.

### Activities

Figure 1 illustrates the logic model of the relationship between the Institute’s resources, activities, and intended effects. Because of the integrated design of the content and process in the program, it is not always feasible to separate curricular content from process. The pedagogical method itself was often the content of the learning and the group of faculty participants purposefully served as its own learning laboratory.

#### Peer mentoring

During the Institute, each participant held the roles of both mentor and protégé. With the entire cohort in a circle, the facilitators explained the exercises and ensured broad participation. Mentoring practices such as listening, affirmation, relationship formation, challenge and support, were modeled by the facilitators. The participants practiced these strategies in their interactions with each other in their small subgroups when working on Academic Development Plan steps and during other content areas.

#### Group formation

Written guidelines to introduce group norms of behavior and to ensure a trustworthy space for participation and confidentiality were discussed at the first session and revisited at the start of each session. Exercises were structured that would intentionally encourage relationship formation among participants.

#### Structured steps in career development: the Academic Development Plan (ADP)

A systematic guided approach to career planning was undertaken by each participant, identifying 1-, 3-, and 10-year professional and personal goals [36]. The 13 steps of the ADP were embedded sequentially throughout all nine Institute days. Participants used worksheets for each step, which were filed into the participant’s personal binder. After a facilitator demonstration, participants were helped by their peers to identify the tasks that would be needed to be undertaken and skills to be learned to attain each of their goals. Participants mentored each other in triads and foursomes on each ADP step, with changing composition of the small groups to ensure that each participant could benefit from receiving and offering mentoring from and to all the other faculty in the cohort, incorporating varied perspectives and expertise. Participants constructed written learning contracts[25] with themselves that included a verification method for the completion of each task or skill and a timeline. Each participant formulated a detailed, coherent written plan to be executed on a clear timeline.

#### Alignment of personal values and career goals

The Institute emphasized values alignment [1,37]. Participants discerned their own core personal values[37] by examining past choices and the motivation of those choices and used this new understanding as a basis to consider the extent of alignment with their current and future professional roles and responsibilities as part of their ADP [36].

#### Strengths identification

Participants discerned their own strengths by examining times when they had been exceptionally successful. They factored this knowledge into their ADP steps, career choices, and planned roles.

#### Skill development

**Figure 2.**
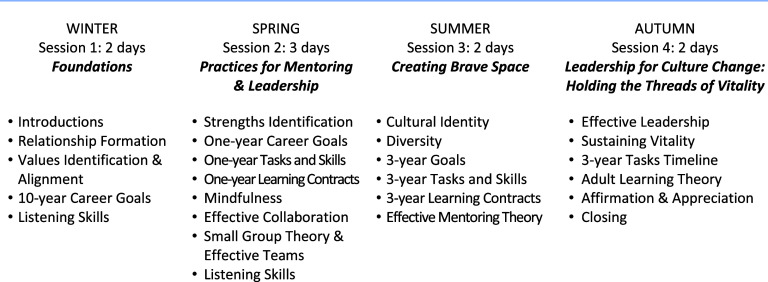
C-Change Mentoring and Leadership Institute curriculum content.

Figure 2 shows the chronology of curricular content and skill development foci. Listening skills were introduced early in the course and were formally taught, practiced at each session, and integrated into different content areas.

#### Reflective practices

Several reflective practices were woven into each day and included a daily check-in as a centering activity.

-Writing: formal reflective writing exercises were incorporated daily (e.g., “What do you find most meaningful in your work?”). Participants wrote at the end of each day to capture important learnings from the day to solidify, clarify, and remember them. Participants assembled their writings in their personal binders.

-Poetry: Seasonal metaphors[39] were augmented by poetry, chosen as relevant to the day’s content areas, and read aloud by the group.

#### Mindfulness

Participants discussed articles that focused on the benefits and practices of mindfulness in biomedicine and how such practices could benefit their roles as physicians and scientists [40–42].

#### Cross-cultural practice and valuing differences

Facilitated exercises to foster awareness and valuing of differences and diversity were built in over the year. The exercises heightened participants’ awareness of their own identities, development of cognitive empathy [43], and focused attention on differences within the cohort and aspects of marginalization. At the third quarterly session, after trusting relationships had been established across the cohort, participants reintroduced themselves using the lens of their cultural heritage, family of origin, and their experiences of feeling different. Listening to the stories deepened participants’ understanding of the ways in which others had experienced marginalization and the influence of those experiences.

#### Effective collaboration and teamwork

Participants described their experiences of professionally working in teams or groups to identify attributes of effective collaboration. Content included the theoretical concepts of how small groups, or teams, behave predictably [29], the tasks and stages of group development, and the roles and responsibilities of an effective team leader.

#### Models of effective leadership

The group discussed key theoretical frameworks of effective leadership. Participants considered their own leadership roles and patterns of leadership behavior. They explored, with guidance from their peers, the perspectives of various stakeholder groups relevant to leading their own change activities in their home institutions.

### C-Change Mentoring and Leadership Institute process

#### Variety of learning modalities

The Institute was designed to include all learners: extrovert and introvert, with prior expertise on a topic or learning about it for the first time. Novel dialog strategies such as World Café [44] and Appreciative Inquiry[45] were used. Each curricular content area (Figure 2) was taught in multiple ways to engage individuals with differing learning styles, backgrounds, and personalities, with a goal of involving everyone in active dialog. Each day started with a check-in period with participants sitting in a circle. The Institute employed a combination of entire group, and small subgroups (in dyads or triads), followed by a reflective practice, usually writing by hand in response to a relevant probing question about the content and its relevance to individual participant’s needs. The facilitators debriefed participants who were encouraged to articulate their own learning and hear about the learning of other participants. Key articles were provided for each content area.

In addition to completing tasks for individual career development, the group simultaneously observed and learned about the culture of the Institute, such as the stages of group development, novel dialog strategies, effective mentoring practices, and affirmative patterns of communication. Electronic devices were highly discouraged in the sessions, and all writing was done by hand.

### Measurable outcomes

Below are the five major measurable outcomes in the logic model: a) amount and quality of mentoring and its key components, b) change in vitality, c) self-efficacy, d) inclusion, and e) valuing diversity and skills for addressing inequity.

#### Amount and quality of mentoring and its key components

Assessment of the amount of mentoring received by participants utilized a six-point response scale ranging from “*None” to “A Lot*.” Satisfaction with the amount of mentoring received used a five-point response scale ranging from “*Very Dissatisfied” to “Very Satisfied*.” A ten-item mentoring quality measure including key components of mentoring [46] was used to assess participants’ views on the Institute’s mentoring helpfulness in accomplishing career advancement, research objectives, and values alignment (Table 2).

**Table 2.** C-Change mentoring quality and key components and inclusion^a^ measures: items, response scale, and internal consistency

Mentoring Quality and Key Components
***To what extent has mentoring in the C-Change Institute helped you …***
formulate your *career* goals formulate your *research* goals plan how to achieve your *career* goals plan how to achieve your *research* goals learn the skills needed to succeed in your *career* goals learn the skills needed to succeed in your *research* goals find the resources you need have a sponsor/champion/network to advance your career or your work plan how to achieve your *personal* goals assess how well your professional activities align with your personal values
*Six-point ordinal extent scale from “Not at all” to “To a Very Large Extent.” Estimated Cronbach alpha coefficient .96.*
**Inclusion**
***My C-Change Institute group …***
gives me the feeling that I belong gives me the feeling that I am part of the group treats me as an insider likes me appreciates me cares about me allows me to be who I am encourages me to be who I am
*Seven-point ordinal agreement scale from “Very Strongly Disagree” to “Very Strongly Agree.” Estimated Cronbach alpha coefficient .96.*

a Inclusion measure adapted from Jansen[47].

#### Change in vitality

Participants reported their own change in vitality from the prior year using a seven-point agreement scale ranging from “*Very Strongly Disagree” to “Very Strongly Agree*,” (four items, alpha .93).

#### Self-efficacy

Self-efficacy was assessed in three domains: a) career advancement – (four items, alpha .83) using a seven-point anchored scale denoting “*Completely False” to “Completely True*;” b) research (four items, alpha .87), and c) work-life integration (five items, alpha .90) both used a six-point confidence scale denoting “*Not at all Confident” to “Completely Confident*.”

#### Inclusion

The degree of inclusion felt by participants in the Institute was assessed by an eight-item measure adapted from Jansen [47], which used a seven-point anchored agreement scale, alpha 0.96 (Table 2).

#### Valuing diversity and skills for addressing inequity

Valuing diversity related to attitudes and behaviors was assessed with a six-point true-false scale, “*Very Untrue -Very True*” (nine items, alpha .89), and agency to address inequity assessed the extent to which mentoring in the Institute helped participants develop the skills to work against: a) racial and ethnic inequity; b) anti-Black racism; and c) gender inequity using a six-point scale ranging from “*Not at all” to “To a Very Large Extent*” (three items, alpha .82).

### Data analysis

Data were analyzed using SAS/STAT Version 9.4 for Windows, 2006 (SAS Institute, Cary, NC). Pearson correlations were calculated for interval scales and t-tests examined differences between demographic groups on the mentoring measure.

This study was approved by Brandeis University Human Subjects Protection (IRB #19127R-E). Written informed consent was provided by all participants for the publication of deidentified data.

## Results

Of the 40 faculty enrolled in the intervention, 35 completed the C-Change Mentoring & Leadership Institute by attending all sessions. Three of the five noncompleting faculty left the program after the initial session due to Covid travel concerns, the expense and burden of transcontinental travel, and two for lack of fit with the program elements. Participants constructed a written ADP and completed the post-intervention evaluation survey.

### Assessment of mentoring

Faculty participants rated highly their experience of mentoring received in the Institute. These perspectives did not differ significantly across gender, race, ethnicity, or degree (Table 3).

**Table 3.** Ratings of C-Change mentoring quality and key components (mean values and 95% confidence interval) for 35 midcareer research faculty completing the C-Change Mentoring and Leadership Institute 2020–2022 by demographic group

Characteristics	Faculty completing Institute (n = 35)	Ratings of mentoring quality	
	Number (%)	Mean	95% CI
Total	35 (100)	4.98	4.64-5.32
Gender			
Female	16 (46)	4.65	4.02-5.27
Male	19 (54)	5.26	4.92-5.60
Race and Ethnicity			
URM	17 (49)	5.28	4.87-5.69
Non-URM	18 (51)	4.70	4.16-5.24
Degree			
M.D. or M.D./Ph.D.	20 (57)	5.16	4.73-5.58
Ph.D.	15 (43)	4.75	4.16-5.34

URM: underrepresented in medicine. Individuals from racial and ethnic groups that have low representation in the health-related sciences and STEM fields on a national basis, as designated by the National Institutes of Health and the National Science Foundation. Non-URM: nonunderrepresented in medicine. National Institutes of Health. November 22, 2019. Accessed July, 2024 https://grants.nih.gov/grants/guide/notice-files/NOT-OD-20-031.html.

Eighty-five percent of participants reported receiving “*a lot*” or “*a considerable amount*” of mentoring and 82% were “*very*” satisfied with the mentoring they received. The remainder was “*somewhat*” satisfied. Regarding the key components of mentoring (Table 2), 88% of participants found the Institute helpful to “*a large or very large extent*” in formulating their career goals and 82% in planning how to achieve those goals. More than two-thirds reported that the Institute was helpful to “*a large or very large extent*” in formulating their research goals (71%), planning how to achieve research goals (67%), and over half in learning the skills needed to succeed in their research goals (59%). Seven in 10 participants reported that the Institute aided them to “*a large or very large extent*” in both learning the skills needed to succeed in their career goals (71%) and in planning how to achieve their personal goals (71%). Eighty-two percent of participants indicated that the Institute had to “*a large or very large extent”* helped them assess how well their professional activities aligned with their personal values. The Institute was less effective (aligned with Institute objectives) in helping participants “find the resources they need” (50% to “a *large/very large extent”)*, or “have a sponsor/champion/network to advance your career or your work” (44%). Even so, it is interesting that the peer group was able to provide these components of mentoring to about half of participants.

### Correlation of mentoring ratings with other study outcomes (Table 4)

High mentoring ratings were positively correlated with better outcomes in self-assessed change in vitality, and self-efficacy in career advancement, research, and work-life integration. Feelings of inclusion were a highly statistically significant and strong predictor of participant-rated mentoring at 0.62, *P* < 0.001. Skills that were developed in the Institute for addressing inequity were highly correlated with mentoring rating at 0.78, *P* < 0.001, and with valuing diversity at 0.45, *P* = 0.008. Analyses revealed no differences in impact between the two cohorts of faculty.

**Table 4.** Correlations of ratings of C-Change mentoring quality and key components with other study outcome variables for 35 midcareer research faculty completing the C-Change Mentoring and Leadership Institute 2020–2022

Measurable Outcome	Correlation with ratings of mentoring quality	
	Pearson correlation coefficient	*P*-value
**Self-assessed change in vitality** *Current vitality compared to 1 year ago*	0.57	<0.001
**Self-efficacy: career advancement** *Self-confidence in ability to advance in career*	0.51	0.002
**Self-efficacy: research** *Self-confidence in ability to be successful in research*	0.37	0.030
**Self-efficacy: work-life integration** *Self-confidence in ability to effectively manage work and personal responsibilities*	0.46	0.005
**Inclusion^a^** *Feelings of belonging, being appreciated, and allowed to be true self*	0.62	<0.001
**Valuing diversity: attitudes and behaviors** *Extent of valuing diversity in work teams, recruitment, and advancement*	0.45	0.008
**Skills for addressing inequity** *Ability to identify and respond to gender, race, and ethnicity inequity*	0.78	<0.001

a Adapted from Jansen [47].

## Discussion

Experienced facilitators implemented a career development curriculum and process based on theoretical foundations that resulted in a positive mentoring experience for the midcareer faculty participants. The Institute increased vitality and fostered career advancement in research faculty; created a framework and environment for effective career guidance and personal awareness; and provided the means to fully include women and persons from groups that are underrepresented in medicine by race, ethnicity and gender issues. The Institute’s design modeled relational behaviors and minimized problems of hierarchy, power differentials, issues of gender, race, and ethnicity discordance, and differences in opinions about the effectiveness of mentoring between mentees and their mentors [48–52]. We were able to show strong correlations between effective mentoring and the measured key outcomes of enhanced faculty vitality, self-efficacy, feelings of inclusion, and cross-cultural awareness and skills.

The structure, content, and process of the intervention were intentionally a culture change paradigm whereby Institute participants experienced learning in an environment that contrasts with that usually experienced in medical institutions where hierarchy and isolation are prevalent [21]. Meetings enacted characteristics of the culture we believe are necessary to support trustworthy relationship formation, align personal core values and career goals, support the humanity of faculty, and include the perspectives and skills of faculty who belong to groups that are underrepresented in medicine.

Academic medicine is populated by faculty who entered medicine with altruism and passion but have often ignored their humanistic inner needs while they have ably undertaken the external demands of their work. The Institute’s curriculum addressed the original inner needs and thus contributed to sustaining the vitality of the research faculty. This is a marked contrast in the curriculum and process of the Institute when compared with traditional faculty development that seldom addresses these inner needs.

Another contrast with customary faculty development practices is the integration of career advancement goals with cross-cultural competence and diversity goals. In the diversity realm, interpersonal connections, sense of inclusion, and psychological safety[53] were key. NIH notes that “there are many benefits that flow from a diverse supported scientific workforce, including fostering scientific innovation, enhancing global competitiveness, contributing to robust learning environments, improving the quality of the research, advancing the likelihood that underserved or health disparity populations participate in, and benefit from health research, and enhancing public trust [54–60].” In addition to these rich rewards, we can add the development of valuing differences[19] and developing skills to address inequity. We share the view of the NIH that a diverse group working together could capitalize on innovative ideas and distinct perspectives [33,34]. This was especially evident in attaining our cross-cultural competence goals. In contrast to many “diversity” faculty development efforts, whose impact has been questioned [61], the Institute process and content did positively impact important cross-cultural competence outcomes.

Our prior research showed that relationship formation and value alignment are positively linked to faculty vitality [1]. Building on this research, we devoted substantial time in the Institute to these two foundational concepts. We believe that excellence and high-faculty vitality can only result if faculty values are aligned with their professional roles and efforts, and that they are functioning in a relational environment where they feel they are included and belong. This is especially important for leaders, who by demonstration of the values they profess and the policies and practices they establish and nurture, cultivate the supportive culture of an organization. We anticipated that many of our faculty participants – as they assume leadership roles in academic medicine – will be able to bring their awareness of their own values to these influential roles.

Limitations of the study included the self-selection of cohorts of already successful faculty, expense of such a program, and it is too soon to determine the duration of intervention impact. It is uncertain how much of the positive intervention effect is attributable to the characteristics of the two facilitators. The stratified demographic diversity in the faculty sample was important and may be challenging to replicate.

## Conclusions

The outcomes of the Institute align with C-Change program theory in predictable ways, and we were able to determine that the Institute works consistently with our model. By experiencing learning in an altered working environment that was intentionally affirming, noncompetitive, trustworthy, relational, and reflective, faculty participants may be able to build a similar culture with colleagues and trainees in their home institutions, thus modeling effective culture change. The correlation of mentoring effectiveness and the inclusion scale assessment suggests a strong relationship between the level of trust achieved among participants and the desired mentoring outcomes. This intervention shifts the widely used dyadic model of mentoring to a facilitated peer group mentoring strategy. Doing so eliminates common pitfalls of one-on-one mentoring, such as power hierarchy and paucity of senior mentors. Although large numbers of women and historically excluded racial and ethnic group members have completed M.D. and Ph.D. training, these groups have experienced more barriers to career advancement [62]. Group peer mentoring could contribute to addressing the gender disparity in medical school leadership [63,64]. The intervention brings attention and training in cultural diversity into the mainstream of career development for all groups of faculty, rather than secluding it in programs specifically addressing the needs of homogeneous groups. We hope this detailed account will provide a way forward to successful mentoring and career development for others.

## Data Availability

The datasets generated and analyzed during this study are not publicly available as these data would allow identification of participating faculty. Individual participant privacy could be compromised due to the small number of participants and their diverse demographic attributes. Subjects signed Brandeis University IRB-approved informed consent forms that stated that their data would be held confidentially and not be shared in the public domain. IRB Contact: (email) hrpp@brandeis.edu, (tel) (781) 736 8133.

## References

[1] Pololi LH , Evans AT , Civian JT , et al. Faculty vitality—surviving the challenges facing academic health centers: a national survey of medical faculty. Acad Med. 2015;90(7):930–936. 25692560 10.1097/ACM.0000000000000674

[2] Pololi LH , Evans AT , Civian JT , et al. Are researchers in academic medicine flourishing? A survey of midcareer Ph.D. and physician investigators. J Clin Trans Sci. 2023;7(1):e105. 10.1017/cts.2023.525 PMC10225255 37251000

[3] Pololi LH , Evans AT , Civian JT , et al. Mentoring faculty: a U.S. national survey of its adequacy and linkage to culture in academic health centers. J Contin Educ Health Prof. 2015;35(3):176–184. 26378423 10.1002/chp.21294

[4] Pololi LH , Krupat E , Civian JT , Ash AS , Brennan RT. Why are a quarter of faculty considering leaving academic medicine? A study of their perceptions of institutional culture and intentions to leave at 26 representative U.S. medical schools. Acad Med. 2012;87(7):859–869. 22622213 10.1097/ACM.0b013e3182582b18

[5] Alexander H , Lang J. The long-term retention and attrition of U.S. medical school faculty. AAMC Analysis in Brief. 2008;8:1–2.

[6] West CP , Dyrbye LN , Shanafelt TD. Physician burnout: contributors, consequences and solutions. J Intern Med. 2018;283(6):516–529. 29505159 10.1111/joim.12752

[7] Hall S. A mental-health crisis is gripping science — toxic research culture is to blame. Nature. 2023;617(7962):666–668. 37221336 10.1038/d41586-023-01708-4

[8] Martinson BC , Anderson MS , De Vries R. Scientists behaving badly. Nature. 2005;435(7043):737–738. 15944677 10.1038/435737a

[9] Rockey S. Retention rates for first-time R01 awardees [Internet]. National Institutes of Health. 2014. Available from: (https://nexus.od.nih.gov/all/2014/10/28/retention-of-first-time-r01-awardees/) Accessed July 5, 2024.

[10] Committee on the Impacts of Sexual Harassment in Academia, Committee on Women in Science, Engineering, and Medicine, Policy and Global Affairs, National Academies of Sciences, Engineering, and Medicine. Sexual harassment of women: Climate, culture, and consequences in academic sciences, engineering, and medicine. Johnson PA , Widnall SE , Benya FF. editors. Washington, D.C: National Academies Press, 2018. Available from: (https://www.nap.edu/catalog/24994) Accessed July 5, 2024. 29894119

[11] Wellcome. What researchers think about the culture they work in. Wellcome. 2020. Available from: (https://wellcome.org/reports/what-researchers-think-about-research-culture) Accessed July 5, 2024.

[12] O’Connor P. Why is it so difficult to reduce gender inequality in male-dominated higher educational organizations? A feminist institutional perspective. Interdiscip Sci Rev. 2020;45(2):207–228.

[13] Clancy KBH , Nelson RG , Rutherford JN , Hinde K. Survey of academic field experiences (SAFE): trainees report harassment and assault. In: Apicella CL , ed. PLOS ONE, 2014:9(7):e102172, 25028932 10.1371/journal.pone.0102172 PMC4100871

[14] Pololi LH , Brennan RT , Civian JT , et al. Sexual harassment within academic medicine in the United States. Am J Med. 2019;133(2):245–248. 31301297 10.1016/j.amjmed.2019.06.031

[15] Woolston C. Discrimination still plagues science. Nature. 2021;600(7887):177–179. 34845341 10.1038/d41586-021-03043-y

[16] Hardeman RR , Homan PA , Chantarat T , Davis BA , Brown TH. Improving the measurement of structural racism to achieve antiracist health policy: study examines measurement of structural racism to achieve antiracist health policy. Health Aff. 2022;41(2):179–186. doi: 10.1377/hlthaff.2021.01489. PMC9680533 35130062

[17] Braveman PA , Arkin E , Proctor D , Kauh T , Holm N. Systemic and structural racism: definitions, examples, health damages, and approaches to dismantling: study examines definitions, examples, health damages, and dismantling systemic and structural racism. Health Aff. 2022;41(2):171–178. doi: 10.1377/hlthaff.2021.01394. 35130057

[18] Crites GE , Ward WL , Archuleta P , et al. A scoping review of health care faculty mentorship programs in academia: implications for program design, implementation, and outcome evaluation. J Contin Educ Health Prof. 2022;43(1):42–51. doi: 10.1097/CEH.0000000000000459. 36215162

[19] Pololi LH , Evans AT , Brimhall-Vargas M , et al. Randomized controlled trial of a group peer mentoring model for U.S. academic medicine research faculty. J Clin Trans Sci. 2023;7(1):e174. doi: 10.1017/cts.2023.589. PMC10465314 37654777

[20] Pololi LH , Evans AT , Civian JT , McNamara T , Brennan RT. Group peer mentoring is effective for different demographic groups of biomedical research faculty: a controlled trial. PLOS ONE. 2024;19(3):e0300043. doi: 10.1371/journal.pone.0300043. 38498502 PMC10947691

[21] Pololi L , Conrad P , Knight S , Carr P. A study of the relational aspects of the culture of academic medicine. Acad Med. 2009;84(1):106–114. 19116486 10.1097/ACM.0b013e3181900efc

[22] National Institutes of Health, NIH Office of Strategic Coordination – The Common Fund. A decade of discovery: The NIH Roadmap and Common Fund. 2014. NIH Pub No. 14-8013. Available from: (https://commonfund.nih.gov/sites/default/files/ADecadeofDiscoveryNIHRoadmapCF.pdf).

[23] Penland P. Self-initiated learning. Adult Educ. 1979;29(3):170–179.

[24] Knowles MS. The Modern Practice of Adult Education: From Pedagogy to Andragogy. New York, NY: The Adult Education Company, 1980.

[25] Brookfield SD. Understanding and Facilitating Adult Learning. San Francisco, CA: Jossey-Bass, 1986.

[26] Rogers CR. The facilitation of significant learning. In: Siegel L , ed. Instructions: Some contemporary viewpoints. San Francisco, CA: Chandler, 1967:37–54.

[27] Yalom ID. The theory and practice of group psychotherapy. 4th ed. Basic Books; 1995.

[28] Tuckman BW. Developmental sequence in small groups. Psychol Bull. 1965;63:384–399. doi: 10.1037/h0022100. 14314073

[29] Ryan RM , Deci EL. Self-determination theory and the facilitation of intrinsic motivation, social development, and well-being. Am Psychol. 2000;55(1):68–78. 11392867 10.1037//0003-066x.55.1.68

[30] Freire P. Pedagogy of the Oppressed. New York, NY: Continuum, 1986.

[31] Notice of NIH’s Interest in Diversity, National Institutes of Health, Office of Extramural Resources, 2019. 2019. (https://grants.nih.gov/grants/guide/notice-files/NOT-OD-20-031.html) Accessed July 5, 2024.

[32] National Institutes of Health, National Institute of General Medical Sciences. Enhancing the diversity of the NIH workforce [Internet]. Bethesda (MD): National Institutes of Health. (https://nigms.nih.gov/training/dpc) [updated 2023 Mar 17; cited 2023 Sep 13]. Accessed July 5, 2024.

[33] Valantine HA , Collins FS. National institutes of health addresses the science of diversity. Proc Natl Acad Sci USA. 2015;112(40):12240–12242. 26392553 10.1073/pnas.1515612112 PMC4603507

[34] Collins FS , Adams AB , Aklin C , et al. Affirming NIH’s commitment to addressing structural racism in the biomedical research enterprise. Cell. 2021;184(12):3075–3079. 34115967 10.1016/j.cell.2021.05.014

[35] Gomez LE , Bernet P. Diversity improves performance and outcomes. J Natl Med Assoc. 2019;111(4):383–392. 30765101 10.1016/j.jnma.2019.01.006

[36] Pololi L. Career development for academic medicine—a nine step strategy. BMJ. 2006;332(7535):s38–s39.

[37] Pololi L , Kern DE , Carr P , Conrad P , Knight S. The culture of academic medicine: faculty perceptions of the lack of alignment between individual and institutional values. J Gen Intern Med. 2009;24(12):1289–1295. 19834773 10.1007/s11606-009-1131-5 PMC2787944

[38] Schmitt MJ , Schwartz S , Steyer R , Schmitt T , Eur J Psychol Assess. 1993;9:107–121.

[39] Palmer PJ. Seasons: A Center for Renewal. Kalamazoo: Fetzer Institute, 1999.

[40] Meier DE. The inner life of physicians and care of the seriously ill. JAMA. 2001;286(23):3007. 11743845 10.1001/jama.286.23.3007

[41] Pearsall P. Toxic success and the mind of a surgeon. Arch Surg. 2004;139(8):879. 15302698 10.1001/archsurg.139.8.879

[42] Epstein RM. Mindful practice. JAMA. 1999;282(9):833. 10478689 10.1001/jama.282.9.833

[43] Reniers R , Corcoran R , Drake R , Shryane NM , Völlm BA. The QCAE: a questionnaire of cognitive and affective empathy. J Pers Assess. 2011;93(1):84–95. 21184334 10.1080/00223891.2010.528484

[44] Brown J , Isaacs D. The World Café: shaping our futures through conversations that matter. 1st ed. San Francisco, CA: Berrett-Koehler Publishers, 2005.

[45] Cooperrider DL. editor.Appreciative Inquiry: Rethinking Human Organizations Toward a Positive Theory of Change. Champaign: Stipes, 2000.

[46] Pololi L , Evans A , Civian J , Gibbs B , Gillum L , Brennan R. A novel measure of “good” mentoring: testing its reliability and validity in four academic health centers. J Contin Educ Health Prof. 2016;36(4):263–268. 28350307 10.1097/CEH.0000000000000114

[47] Jansen WS , Otten S , van der Zee KI , Jans L. Inclusion: conceptualization and measurement. Eur J Soc Psychol. 2014;44(4):370–385.

[48] Straus SE , Chatur F , Taylor M. Issues in the mentor-mentee relationship in academic medicine: a qualitative study. Acad Med. 2009;84(1):135–139. 19116493 10.1097/ACM.0b013e31819301ab

[49] Hansman CA. Diversity and power in mentoring relationships, critical perspectives on mentoring: trends and issues. In: Hansman C , Mott V , Ellinger AD , Guy T , eds. Eric Info Series No. 388. Columbus (OH): ERIC Clearinghouse on adult, career, and vocational education, Ohio State University, 2002: 39–48.

[50] Lagay F. If role models do model roles. AMA J Ethics. 2002;4(11):329–330. 10.1001/virtualmentor.2002.4.11.jdsc1-0211 23268751

[51] Pololi L , Knight S. Mentoring faculty in academic medicine: a new paradigm? J Gen Intern Med. 2005;20(9):866–870. 16117759 10.1111/j.1525-1497.2005.05007.x PMC1490198

[52] Tung J , Kuiava M , Vuong S , Zhang W , Mancuso C , Salmon J. Lack of reciprocation in the reported relationships between mentors and mentees on the annual review: an opportunity to Formalize mentoring. J Faculty Development. 2024;38(2):53–59.

[53] Laird L , Bloom-Feshbach K , McNamara T , Gibbs B , Pololi L. Psychological safety: creating a transformative culture in a faculty group peer-mentoring intervention. TCOM&C. 2024;8(1):127–140. doi: 10.62935/hz7383. PMC11360255 39210949

[54] Plan for Enhancing Diverse Perspectives (PEDP), National Institutes of Health, The BRAIN Initiative. (https://braininitiative.nih.gov/vision/plan-enhancing-diverse-perspectives) Accessed July 24, 2024.

[55] Valantine HA , Lund PK , Gammie AE. From the NIH: a systems approach to increasing the diversity of the biomedical research workforce. CBE Life Sci Educ. 2016;15(3):fe4. doi: 10.1187/cbe.16-03-0138. PMC5008902 27587850

[56] Science benefits from diversity. *Nature*. 2018;558(7708):5–5. doi: 10.1038/d41586-018-05326-3. 31076730

[57] Freeman RB , Huang W. Collaboration: strength in diversity. Nature. 2014;513(7518):305–305. doi: 10.1038/513305a. 25230634

[58] Hofstra B , Kulkarni VV , Munoz-Najar Galvez S , He S , Jurafsky B , McFarland DA. The diversity-innovation paradox in science. Proc Natl Acad Sci USA. 2020;117(17):9284–9291. doi: 10.1073/pnas.1915378117. 32291335 PMC7196824

[59] AlShebli BK , Rahwan T , Woon WL. The preeminence of ethnic diversity in scientific collaboration. Nat Commun. 2018;9(1):5163. doi: 10.1038/s41467-018-07634-8. 30514841 PMC6279741

[60] Yang Y , Tian TY , Woodruff TK , Jones BF , Uzzi B. Gender-diverse teams produce more novel and higher-impact scientific ideas. Proc Natl Acad Sci USA. 2022;119(36):e2200841119. doi: 10.1073/pnas.2200841119. 36037387 PMC9456721

[61] Singal J. What if diversity trainings do more harm than good?: op-ed. *The New York Times*. 2023;Section SR:9.

[62] Ginther DK , Schaffer WT , Schnell J , et al. Race, ethnicity, and NIH research awards. Science. 2011;333(6045):1015–1019. 21852498 10.1126/science.1196783 PMC3412416

[63] Abdellatif W , Ding J , Jalal S , et al. Leadership gender disparity within research-intensive medical schools: a transcontinental thematic analysis. J Contin Educ Health Prof. 2019;39(4):243–250. 31633570 10.1097/CEH.0000000000000270

[64] Valantine HA. Where are We in bridging the gender leadership gap in academic medicine? Acad Med. 2020;95(10):1475–1476. doi: 10.1097/ACM.0000000000003574. 32639260

